# Seasonal changes in suicide in South Korea, 1991 to 2015

**DOI:** 10.1371/journal.pone.0219048

**Published:** 2019-06-28

**Authors:** Chi Ting Yang, Paul S. F. Yip, Eun Shil Cha, Yi Zhang

**Affiliations:** 1 Department of Statistics and Actuarial Science, The University of Hong Kong, Hong Kong; 2 Centre for Suicide Research and Prevention, The University of Hong Kong, Hong Kong; 3 Department of Social Work and Social Administration, The University of Hong Kong, Hong Kong; 4 Department of Preventive Medicine, Korea University College of Medicine, Seoul, South Korea; 5 Institute of Population and Labor Economics, The Chinese Academy of Social Sciences, Bejing, People’s Republic of China; Kurdistan University of Medical Sciences, ISLAMIC REPUBLIC OF IRAN

## Abstract

Seasonality of suicidal behavior has been widely reported in many epidemiological studies with a well replicated suicide peak in spring followed by a trough in winter season. Research from some regions over the past few decades has shown a diminishing seasonal pattern of suicides and this introduced a new perspective on the suicide study. Data on all suicide deaths from the period 1991 to 2015 was extracted from the South Korean National Death Registration data set which was made available by Statistics Korea. Our findings confirmed a strong seasonal effect of suicides in South Korea throughout the study period and a marked diminishing pattern was observed since the period of 2006–2010. The rhythm of suicides kept changing across the time intervals with a spring peak followed by a second peak in late summer/autumn. The seasonality varied across age groups and the seasonal effect among the Korean elderly suicides was still found to be significant though a diminishing pattern was observed recently.

## Introduction

Seasonality of suicidal behavior has widely been reported in many epidemiological studies with a well replicated suicide peak in spring followed by a trough in winter season [1–4]. Discrepancy of seasonal rhythm could be observed from the death of suicides’ demographic profile, biological/medical background, social and economic perspectives [1, 3–4]. Chew and McCleary [5] evaluated the influence of social and bioclimatic factors by using 28 countries cross-sectional time series data and reported those populations in the temperature zone exhibiting suicide seasonality. Woo *et al*. [4] studied those articles published from 1979 to 2011 regarding the seasonal variation of suicide rates and summarized a highly replicated peak in spring time. Christodoulou *et al*. [3] reviewed seasonality of suicide studies in both Northern and Southern hemisphere countries and noted most of them reporting seasonality in spring peak and early summer. Also, a similar finding could be generalized to South Africa, Australia and some Asian countries [3, 6–8]. There were some studies, however, reporting no evidence of seasonal variation in suicide mortality [9–13].

It was interesting that research from some regions over the past few decades had shown a diminishing seasonal pattern of suicides and this introduced a new perspective on the suicide study [2]. Yip *et al*. [10] reported a great diminished seasonal effect by examining suicide data in Australia and New Zealand. The decreasing pattern could also be found in some countries like England and Wales, Switzerland and Slovenia [11–12, 14]. Ajdacic-Gross *et al*. [2] conducted a qualitative review on articles published since the 1990s and noted that the seasonality of suicides was tending to diminish and may, eventually, disappear in Western countries. Recently, Kwok and Yip [15] investigated self-harm patients obtained from public hospitals in Hong Kong reporting a great magnitude diminishing in seasonality.

The mortality of suicide remained a significant cause of death in many countries that belong to the Organization for Economic Cooperation and Development (OECD) and the situation also applied to South Korea. In 2015, the estimated suicide rate in South Korea was 28.3 per 100,000 ranking one of the highest rates in the world and also in the top among the OECD members [16]. Many studies tried to depict the myth of high suicide rate in South Korea from their demographic profile, social and cultural perspectives and some cohorts have been reported to be more vulnerable to suicidal behavior [17–19]. Study that reported seasonality of suicide in South Korea was somehow sporadic. Jee *et al*. [20] explored the association of suicide seasonality with solar radiation in the country where a peak of suicide rate in May was reported. However, the baseline characteristic of suicide seasonality and their pattern changed across time intervals have not been well explored.

The aim of this paper is to: a) confirm the presence of seasonal variation in suicide throughout the study period, b) monitor the changes of seasonal pattern in suicides across time intervals, c) whether the diminishing pattern of suicide seasonality can be generalized to a developed Asian society like South Korea are our prime concern.

## Materials and methods

Data on suicide deaths from the period 1991 to 2015 was extracted from the South Korean National Death Registration data set which was made available by Statistics Korea (codes X60–X84, ICD-10). The Statistics Korea collected data from the death notification that was filed at local administration offices together with the death medical certificate issued by physicians [21]. The cause of death was then categorized in accord to the tenth revision of the International Statistical Classification of Diseases and Related Health Problem (ICD-10) that provided a reliable source of official statistics [21]. We also obtained official population estimates from Statistics Korea where the data was stratified by age (5-year age band), sex and administrative districts.

The study period was subdivided into five equal time intervals, 1991 through 1995, 1996 through 2000, 2001 through 2005, 2006 through 2010, and 2011 through 2015 to examine the baseline characteristics of seasonal pattern of suicide at different time intervals. The breakdown allowed us to trace and monitor suicide rhythm across the time intervals considered.

The seasonal variation of suicides was examined in three ways. First, the monthly distribution of suicides for the five intervals was plotted. Second, a chi-square test statistic was applied to examine the evenness of the monthly average suicides for the time intervals considered. Third, a harmonic time series model was employed [10, 22]. It was assumed the monthly suicide incidence as an independent variable that followed a Poisson distribution. Total variance of the distribution of the monthly suicide data could be decomposed into three components: random, seasonal and non-seasonal; where the seasonal variation consisted of components with cycles that repeated themselves an exact number of time each year. The attributed percentage of each component could then be calculated accordingly to account the seasonal variation. The alternative hypotheses assumed the variation of the non-parametric model was purely random for each time interval considered. A detailed description of harmonic analysis and the corresponding significance testing for different components could be found in Pocock (1974) [22].

For the second part of the analysis, the death of suicide was further classified into six subgroups according to their age: under 25, 25–34, 35–44, 45–54, 55–64 and 65+. We explored the seasonal pattern of suicide by age group and their corresponding significance at different time interval was recorded as well.

In our study, the time horizon has been broken down into five equal time intervals where the mean for the Poisson process may vary across the time intervals considered. Therefore, a ratio of seasonal component of variance to random component of variance that being proportional to the overall mean was constructed. The adjusted ratio allowed us to make a robust comparison the importance of seasonal variation in two or more different time periods. An arbitrary but fixed average weekly frequency (μ) was used to correct the existence of mean difference among subgroups; μ = 500 being applied in our study. A greater value of adjusted ratio implied a relatively significant seasonal variation at the time interval [22].

The analysis was conducted using the SAS version 9.4 where the 5%, 1% and 0.1% level of significance were considered in our study.

## Results

### Descriptive statistics

A total of 243,470 suicides were identified in South Korea for the period 1991–2015.

**Fig 1:**
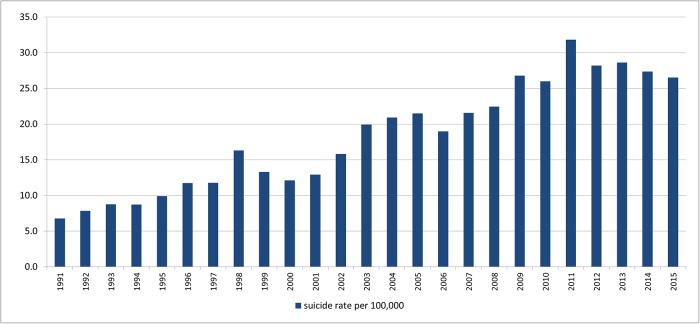
The standardized suicide rate (per 100,000) in South Korea 1991–2015.

Fig 1 gives the overall standardized suicide rate per 100,000 in South Korea annually. A remarkable increment of suicide rate was observed throughout the study period (*p*-value<0.001), climbing from the early rate of 6.8 per 100,000 to the peak 31.8 per 100,000 in 2011. The suicide rate started to fall in the recent time interval of 2011–2015.

**Fig 2:**
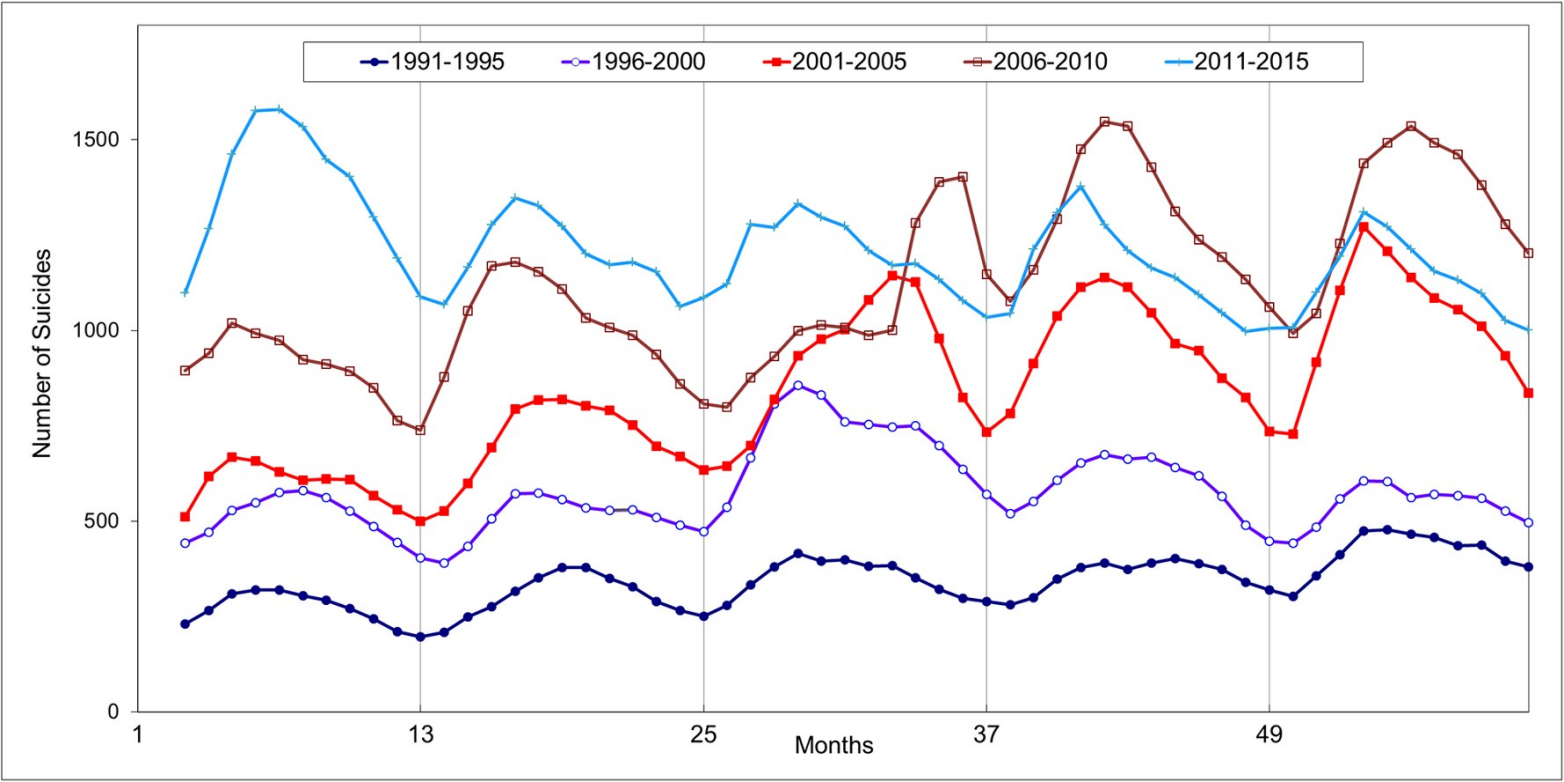
Three-month moving average of suicides in South Korea 1991 to 2015.

**Fig 3:**
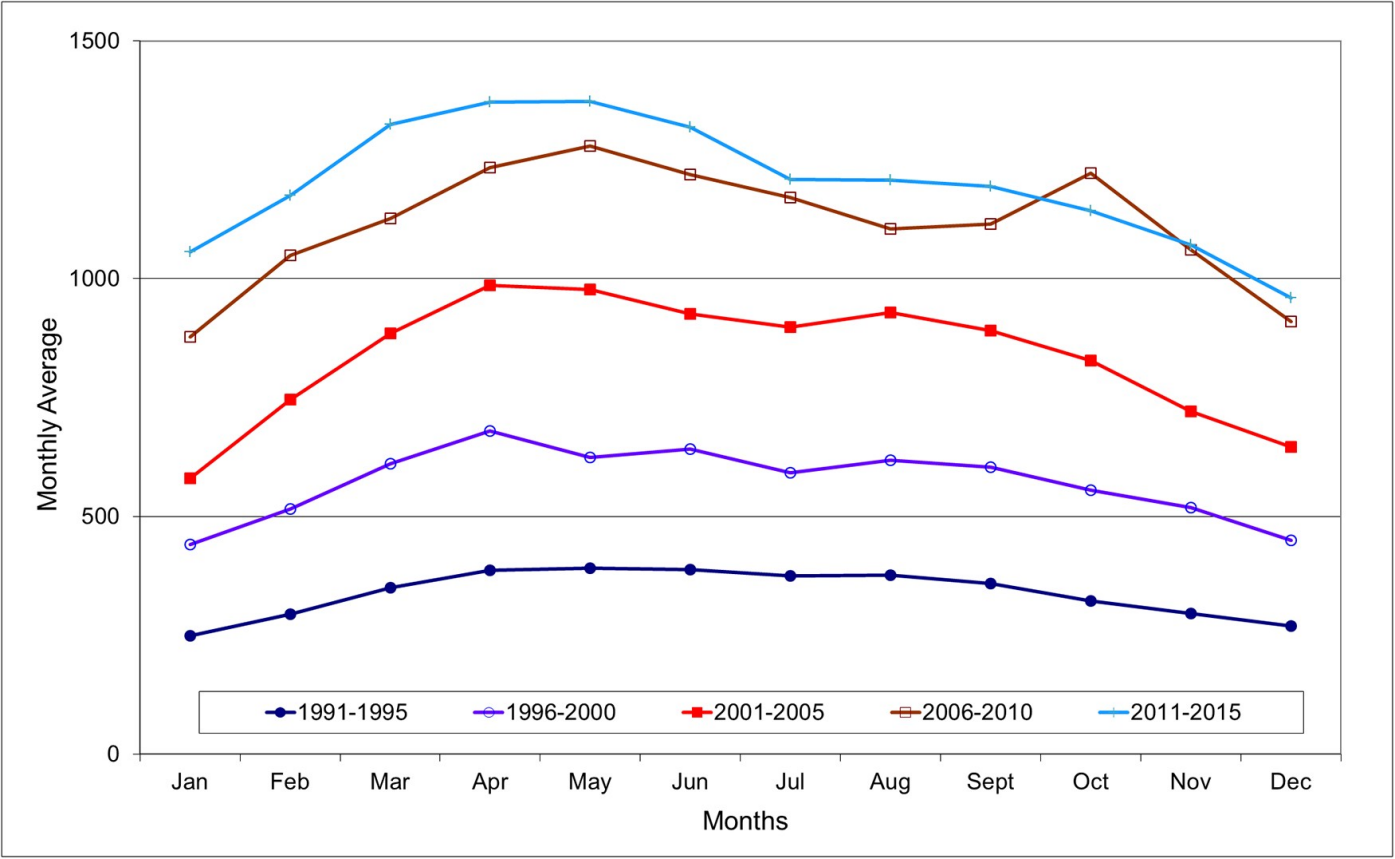
Average monthly distribution of suicides in South Korea 1991–2015.

Fig 2 gives a three-month moving average of suicides in South Korea by every five-year time interval. A systematic cyclic pattern was observed annually, and the pattern became more fluctuated in the recent three intervals. Fig 3 shows the average monthly distribution of suicides by every five-year time interval where the corresponding calendar difference was adjusted. It was noted that the monthly distribution of the first two intervals were rather simple with a general spring peak (April/May), while a relatively low incidence was observed in December and January amongst. For the intervals of 2001–2005 and 2006–2010, the monthly distribution varied greatly with a first spring peak followed by another peak in late summer/autumn. The recent interval (2010–2015) resumed a simple pattern with a peak in April and May, and a trough in winter period. The uneven monthly distribution of the five intervals was supported by the chi-square tests shown in Table 1. Our results confirmed the presence of monthly variation of suicides at different time intervals and the well replicated spring peak was noted in South Korea. The finding agreed with the reporting by Jee *et al*. [20] on suicide study in South Korea as well other literatures of suicide seasonality worldwide.

**Table 1:** The observed and expected number of suicides by month in South Korea 1991–2015 with calendar difference adjusted.

Month	1991–1995		1996–2000		2001–2005		2006–2010		2011–2015	
	Observed	Expected	Observed	Expected	Observed	Expected	Observed	Expected	Observed	Expected
Jan	249	345	441	581	580	850	878	1135	1057	1223
Feb	293	313	513	533	742	773	1046	1033	1169	1112
Mar	350	345	611	581	886	850	1127	1135	1325	1223
Apr	388	333	681	563	986	822	1235	1099	1372	1183
May	391	345	625	581	977	850	1280	1135	1373	1223
Jun	388	333	642	563	927	822	1219	1099	1319	1183
Jul	375	345	592	581	899	850	1172	1135	1210	1223
Aug	377	345	619	581	929	850	1105	1135	1207	1223
Sept	359	333	605	563	892	822	1115	1099	1195	1183
Oct	323	345	555	581	828	850	1223	1135	1143	1223
Nov	296	333	518	563	721	822	1061	1099	1071	1183
Dec	270	345	450	581	646	850	911	1135	961	1223
Chi-Square	81.5		115.7		231.4		162.0		170.8	
*p*-value	<0.001		<0.001		<0.001		<0.001		<0.001	

### Harmonic analysis

Table 2 gives the results of harmonic analysis and the corresponding proportion of variances that were explained by random, seasonal and non-seasonal components by every five-year time interval. For the first interval of 1991–1995, about 76% of the total variance could be explained by the seasonal component and a bi-seasonal pattern was detected (p-value<0.001). The first harmonic cycle (91%) almost dominated in this interval and, in short, an overall one-cycle pattern was concluded. In the meantime, about 13% and 11% of the total variance were attributed to the non-seasonal and random components, respectively. The proportion explained by seasonal component in the second interval (1996–2000) was the same (76%), and the first two harmonic cycles were found to be significant in explaining the seasonal variation. This implied a bi-seasonal model for suicides in this time interval.

**Table 2:** Harmonic analysis of monthly distribution of suicides in South Korea 1991–2015.

Components of Variance	1991–1995	1996–2000	2001–2005	2006–2010	2011–2015
**All seasonal harmonics**	**2293.1 (76%)**	**5267.2 (76%)**	**16148.7 (70%)**	**14487.1 (41%)**	**15704.8 (69%)**
one cycle	2081.4 (91%)***	3933.5 (75%)***	12721.1 (79%)***	8737.8 (60%)***	12958.3 (83%)***
two cycle	203.6 (9%)***	1147.9 (22%)***	3167.3 (20%)***	4409.9 (30%)***	2549.8 (16%)***
three cycle	6.6 (0%)	35.0 (1%)	68.3 (0%)*	893.4 (6%)***	98.5 (1%)*
four cycle	1.5 (0%)	0.0 (0%)	45.6 (0%)	197.6 (1%)**	0.0 (0%)
five cycle	0.0 (0%)	96.2 (2%)**	6.4 (0%)	111.9 (1%)*	98.3 (1%)*
six cycle	0.0 (0%)	54.6 (1%)*	140.0 (1%)**	136.5 (1%)**	0.0 (0%)
**Non-seasonal harmonics**	**391.0 (13%)**	**1126.0 (16%)**	**6012.0 (26%)**	**19653.5 (56%)**	**5807.6 (26%)**
**Random variation**	**338.3 (11%)**	**570.9 (8%)**	**834.4 (4%)**	**1114.1 (3%)**	**1200.1 (5%)**
**Total variance**	**3022.3 (100%)**	**6964.1 (100%)**	**22995.0 (100%)**	**35254.7 (100%)**	**22712.6 (100%)**

*** *p*<0.001

** *p*<0.01

* *p*<0.05

The variance that could be explained by seasonal component dropped for the recent two intervals. The corresponding proportion was about 70% for the interval of 2001–2005 and even lower to 41% for the interval followed by. A greater monthly variation was noted in the interval 2006–2010 where all six harmonic cycles were found to be significant. By comparing with previous intervals, the magnitude of seasonal component for the period has not shown remarkable diminishing and this implied the seasonal effect of suicides was still found to be strong. The proportion of the seasonal component rebounded in the interval of 2011–2015. About 69% of the total variance was accounted by seasonal component where the first two harmonic cycles were found to be significant in explaining the monthly variation. Random component was somehow negligible throughout the study period and the corresponding proportion varied from 3% to 11% of the total variance.

Our study divided the period into five equal time intervals that allowed us to examine the baseline characteristic of suicide seasonality at different time frame. The variation observed between intervals confirmed the movement of seasonal pattern in suicides.

### Seasonality by age group

Suicide deaths were further classified into six subgroups according to their age: under 25, 25–34, 35–44, 45–54, 55–64 and 65+. Table 3 summarizes the harmonic analysis and the proportion of suicide variance that explained by the three components with respect to the deceased’s age group. For the interval of 1991–1995, about 45% of the total variance among the youth and young adults (aged below 35) could be explained by the seasonal component and the seniors (aged 35 and above) the seasonal proportion was over 50% in general. The bi-seasonal pattern of suicides in this interval was mainly contributed by those aged 35–44, 45–54 and the elderly aged 65+, and one-cycle harmonic applied to the rest subgroups.

**Table 3:** Harmonic analysis of monthly distribution of suicides by age group in South Korea 1991–2015.

1991–1995	Under 25	25–34	35–44	45–54	55–64	65+
**All seasonal harmonics**	**68.4 (45%)**	**103.8 (44%)**	**79.7 (56%)**	**62.6 (56%)**	**45.8 (51%)**	**45.4 (54%)**
one cycle	63.1 (92%)***	102.0 (98%)***	68.1 (85%)***	42.6 (68%)***	43.7 (95%)***	38.1 (84%)***
two cycle	0.9 (1%)	0.0 (0%)	9.4 (12%)**	20.0 (32%)***	1.9 (4%)	7.3 (16%)**
three cycle	0.1 (0%)	0.0 (0%)	0.0 (0%)	0.0 (0%)	0.2 (0%)	0.0 (0%)
four cycle	0.0 (0%)	1.8 (2%)	0.5 (1%)	0.0 (0%)	0.0 (0%)	0.0 (0%)
five cycle	0.0 (0%)	0.0 (0%)	0.0 (0%)	0.0 (0%)	0.0 (0%)	0.0 (0%)
six cycle	4.3 (6%)	0.0 (0%)	1.7 (2%)	0.0 (0%)	0.0 (0%)	0.0 (0%)
**Non-seasonal harmonics**	**15.0 (10%)**	**47.4 (20%)**	**0.0 (0%)**	**0.1 (0%)**	**7.3 (8%)**	**0.0 (0%)**
**Random variation**	**67.1 (45%)**	**82.9 (35%)**	**63.8 (44%)**	**49.4 (44%)**	**35.9 (40%)**	**39.2 (46%)**
**Total variance**	**150.6 (100%)**	**234.2 (100%)**	**140.3 (100%)**	**112.1 (100%)**	**89.0 (100%)**	**74.7 (100%)**
**1996–2000**						
**All seasonal harmonics**	**96.8 (53%)**	**153.7 (57%)**	**175.4 (44%)**	**94.7 (38%)**	**190.8 (65%)**	**288.0 (69%)**
one cycle	47.9 (50%)***	141.5 (92%)***	132.9 (76%)***	49.4 (52%)***	140.5 (74%)***	213.5 (74%)***
two cycle	28.4 (29%)***	0.0 (0%)	32.5 (19%)***	40.1 (42%)***	50.4 (26%)***	66.7 (23%)***
three cycle	5.2 (5%)	0.0 (0%)	6.7 (4%)	0.0 (0%)	0.0 (0%)	3.5 (1%)
four cycle	4.6 (5%)	0.0 (0%)	0.0 (0%)	5.1 (5%)	0.0 (0%)	0.0 (0%)
five cycle	7.5 (8%)*	8.6 (6%)*	3.3 (2%)	0.0 (0%)	0.0 (0%)	0.0 (0%)
six cycle	3.1 (3%)	3.5 (2%)	0.0 (0%)	0.0 (0%)	0.0 (0%)	4.3 (2%)
**Non-seasonal harmonics**	**9.1 (5%)**	**0.0 (0%)**	**92.8 (23%)**	**68.0 (27%)**	**27.0 (9%)**	**43.9 (11%)**
**Random variation**	**77.9 (42%)**	**114.7 (43%)**	**128.2 (32%)**	**88.3 (35%)**	**76.3 (26%)**	**85.5 (20%)**
**Total variance**	**183.7 (100%)**	**262.5 (100%)**	**396.5 (100%)**	**251.0 (100%)**	**294.2 (100%)**	**417.4 (100%)**
**2001–2005**						
**All seasonal harmonics**	**120.0 (44%)**	**252.8 (43%)**	**495.8 (51%)**	**388.9 (52%)**	**377.4 (58%)**	**2020.7 (80%)**
one cycle	77.3 (64%)***	196.5 (78%)***	402.0 (81%)***	276.1 (71%)***	292.8 (78%)***	1398.3 (69%)***
two cycle	28.9 (24%)***	46.5 (18%)***	82.5 (17%)***	56.2 (14%)***	80.5 (21%)***	556.0 (28%)***
three cycle	1.8 (1%)	8.2 (3%)*	0.0 (0%)	10.6 (3%)*	0.0 (0%)	3.9 (0%)
four cycle	7.7 (6%)**	1.6 (1%)	4.1 (1%)	1.6 (0%)	0.0 (0%)	0.0 (0%)
five cycle	1.9 (2%)	0.0 (0%)	7.2 (1%)	26.9 (7%)**	4.1 (1%)	8.3 (0%)
six cycle	2.3 (2%)	0.0 (0%)	0.0 (0%)	17.4 (4%)*	0.0 (0%)	54.3 (3%)***
**Non-seasonal harmonics**	**91.2 (33%)**	**221.1 (37%)**	**322.0 (33%)**	**210.4 (28%)**	**149.5 (23%)**	**280.0 (11%)**
**Random variation**	**62.6 (23%)**	**120.8 (20%)**	**163.8 (17%)**	**148.7 (20%)**	**125.0 (19%)**	**213.4 (8%)**
**Total variance**	**273.8 (100%)**	**594.7 (100%)**	**981.5 (100%)**	**748.0 (100%)**	**651.9 (100%)**	**2514.1 (100%)**
**2006–2010**						
**All seasonal harmonics**	**92.8 (19%)**	**251.0 (15%)**	**195.1 (12%)**	**382.0 (36%)**	**288.0 (46%)**	**2770.6 (79%)**
one cycle	16.1 (17%)***	72.4 (29%)***	79.6 (41%)***	183.3 (48%)***	203.3 (71%)***	2073.8 (75%)***
two cycle	32.9 (35%)***	72.0 (29%)***	46.7 (24%)***	157.5 (41%)***	76.3 (27%)***	681.6 (25%)***
three cycle	20.8 (22%)***	40.3 (16%)***	30.5 (16%)**	41.2 (11%)**	4.3 (1%)	11.4 (0%)
four cycle	23.0 (25%)***	18.1 (7%)*	29.4 (15%)**	0.0 (0%)	0.8 (0%)	0.0 (0%)
five cycle	0.0 (0%)	19.4 (8%)*	9.0 (5%)	0.0 (0%)	1.9 (1%)	0.0 (0%)
six cycle	0.0 (0%)	28.8 (11%)**	0.0 (0%)	0.0 (0%)	1.3 (0%)	3.8 (0%)
**Non-seasonal harmonics**	**316.9 (65%)**	**1303.6 (76%)**	**1256.9 (76%)**	**457.6 (43%)**	**185.8 (30%)**	**434.0 (12%)**
**Random variation**	**76.1 (16%)**	**162.5 (9%)**	**193.3 (12%)**	**216.3 (20%)**	**152.7 (24%)**	**313.3 (9%)**
**Total variance**	**485.9 (100%)**	**1717.1 (100%)**	**1645.4 (100%)**	**1055.9 (100%)**	**626.5 (100%)**	**3517.9 (100%)**
**2011–2015**						
**All seasonal harmonics**	**74.1 (53%)**	**207.0 (36%)**	**309.0 (41%)**	**502.6 (56%)**	**361.3 (56%)**	**2439.1 (77%)**
one cycle	40.3 (54%)***	201.5 (97%)***	263.9 (85%)***	457.8 (91%)***	221.1 (61%)***	1853.8 (76%)***
two cycle	24.3 (33%)***	0.0 (0%)	42.2 (14%)**	29.6 (6%)*	102.3 (28%)***	525.9 (22%)***
three cycle	3.4 (5%)	1.5 (1%)	0.0 (0%)	11.4 (2%)	0.0 (0%)	21.8 (1%)
four cycle	0.0 (0%)	0.0 (0%)	0.0 (0%)	0.0 (0%)	0.0 (0%)	4.8 (0%)
five cycle	1.1 (2%)	0.0 (0%)	0.8 (0%)	3.8 (1%)	0.0 (0%)	10.7 (0%)
six cycle	5.0 (7%)*	4.1 (2%)	2.1 (1%)	0.0 (0%)	37.9 (10%)***	22.1 (1%)
**Non-seasonal harmonics**	**0.0 (0%)**	**212.34 (37%)**	**232.06 (31%)**	**142.78 (16%)**	**90.14 (14%)**	**394.55 (12%)**
**Random variation**	**66.7 (47%)**	**156.8 (27%)**	**211.1 (28%)**	**249.0 (28%)**	**188.7 (29%)**	**327.9 (10%)**
**Total variance**	**129.5 (100%)**	**576.1 (100%)**	**752.1 (100%)**	**894.4 (100%)**	**640.2 (100%)**	**3161.5 (100%)**

*** *p*<0.001

** *p*<0.01

* *p*<0.05

For the intervals 1996–2000 and 2001–2005, a bi-seasonal pattern dominated almost in all age groups. Except the elderly aged 65+, the proportion of seasonal component to the total variance dropped in the interval of 2006–2010. It was noted that two-cycle pattern still applied to those aged 55–64 and 65+ for the period while the rest experienced greater temporal fluctuation than that of previous intervals. The proportion of seasonal components rose back for the interval of 2010–2015. The significance of two-cycle pattern in the interval was mainly contributed by those aged below 25, 55–64 and 65+ while one-cycle pattern reported per year among 25–34 and 45–54 subgroups. It was noted that the elderly aged 65+ recorded a relatively higher proportion of seasonality than that of other subgroups since the period of 1996–2000. Our study confirmed the discrepancy of seasonal pattern among age groups and this provided a supplement in elucidating the seasonality of suicide in South Korea.

### Adjusted ratio

The adjusted ratio of seasonal to random components compared the significance of seasonality between intervals. A relatively strong seasonal effect of suicide was noted in the intervals of 1991–1995, 1996–2000 and 2001–2005 with the overall adjusted ratios of 10.2, 8.3 and 10.5 correspondingly (Table 4). The adjusted ratio of suicides dropped in the interval of 2006–2010 (6.1) and even lower to 5.8 in the interval followed by. The recent relatively low values of adjusted ratio suggested that the seasonal effect was playing a less important role in explaining the monthly variation.

**Table 4:** The adjusted ratio of seasonal to random components in suicide by age group in South Korea 1991–2015.

Time Interval	Overall	Under 25	25–34	35–44	45–54	55–64	65+
1991–1995	10.2	7.5	7.6	10.0	12.9	17.8	15.0
1996–2000	8.3	8.1	5.8	5.4	6.2	16.4	20.2
2001–2005	10.5	15.3	8.9	9.2	8.7	12.2	23.4
2006–2010	6.1	7.8	5.1	2.6	4.2	6.3	14.0
2011–2015	5.8	8.5	4.3	3.5	4.0	5.1	11.7

Average weekly frequency (μ = 500)

The adjusted ratio by age group is also given in Table 4. The significance of seasonality in the interval of 1991–1995 was mainly contributed by the seniors aged 45–54, 55–64 and 65+. For the intervals 1996–2000 and 2001–2005, those seniors aged 55–64 and 65+ recorded a relatively higher value of adjusted ratio than that of other subgroups. Youth aged below 25 in the interval of 2001–2005 reported a stronger seasonal effect (adjusted ratio 15.3) by compared with other intervals. Though a diminishing pattern was noted recently, the seasonality among the elderly aged 65+ was still found to be significant by compared with other subgroups (the intervals 2006–2010 and 2011–2015).

The adjusted ratio corrected the discrepancy of mean difference in two or more different time periods and the figure was more robust for the seasonality comparison. Our results suggested a declining trend of suicide seasonality in recent intervals and confirmed the diminishing pattern could be generalized to South Korea. It was noted that the elderly subgroup was the key contributor to the temporal fluctuation of Korean suicides throughout the study period.

## Discussion

Previous studies showed great seasonal variations in the death of suicide and the phenomena also applied to South Korea. This study was the first that extensively examined the seasonal pattern of suicide in South Korea by different time interval using the national data from Statistics Korea, 1991–2015. Our findings confirmed a strong seasonal effect of suicides in South Korea throughout the study period and a marked diminishing pattern was observed since the time interval of 2006–2010. It was noted that the seasonal pattern varied across the time intervals with a well replicated spring peak followed by a second peak in late summer/autumn sometimes. Discrepancy of seasonality could be observed among age groups. The elderly showed a substantial increase in the suicide rate over the study period and, in the meantime, was the key contributor toward the temporal fluctuation in South Korea.

Chew and McCleary [5] examined the influence of social and bioclimatic factors by using 28 countries time series data and reported only those populations in the temperature zone exhibiting suicide seasonality. The climate in the temperate zone always showed the widest seasonal change with longest day length in spring time/early summer that may introduce a potential influence from geographic perspective towards the seasonal cycle of suicides [4]. The activities of serotonin that regulated mood and impulse control of human being was sensitive to the climate change and a marked seasonal fluctuation was reported [23]. It was noted that the malfunctioning of one’s serotonin could be one of the most imperative factors that drive a person to have a deliberate self-harm behavior [24–26]. Therefore, the inter-correlation among the climate change, the functionality of serotonin and suicidal ideation/behavior may somehow help explain the seasonal effect in suicides. In fact, a number of studies have demonstrated that a high correlation between climatic factors particularly in sunshine exposure and the incidence of suicidal behavior [1, 4, 27–28]. Ajdacic-Gross *et al*. [12] further suggested the diminishing in seasonal pattern was mainly attributed to the decline in the agricultural work force that required more/longer sunshine exposure.

The climate attributed greatly to the variation of suicide rate in temperature zone but that may not be the case for those countries located in the tropical zone where the climate shows no marked seasons in the region. No evidence in seasonal pattern was observed in Colombia suicide deaths, the country locating in the intertropical zone with a stable temperature all year long [13]. A similar finding was reported in the city of Sao Paulo which is a municipality in the southeast region of Brazil [29] and in Singapore, one of the Asian countries lying one degree north of the equator [30]. It was interesting that another study by Cantor *et al*. [31] examined Caucasian population living relatively close to the equator in Australia but reported a spring/early summer peak among male suicides and an autumn trough in females. It seemed the influence of climate factor diminished in tropical zone though some degree of variations was reported.

Nayha and Micciolo *et al*. [32–33] suggested the phenomenon may be due to the seasonal variation in communal and social activities. Adverse impacts arising from some unexpected accidents or disasters would also alter the pattern of suicide epidemic [34–36]. In South Korea, many people lost their job and had financial difficulties in the Asian economic crisis 1997–1998, and suicide rate abruptly greatly increased [37]. A chain of celebrity suicide in 2005–2009 led imitative suicidal behavior in the country and the number of suicides from carbon monoxide poisoning rapidly increased after a celebrity’s death in September 2008 [38]. The incident of Sewol ferry that occurred in April 2014 triggered the prevalence of community mental disorder in Ansan area to increase [36]. It was estimated that more than 2 million of Korean people was suffering from mental illnesses annually but only around 15% of them would receive proper treatment [39]. The perceived negative stigma against the treatment of mental illness among the Korean society was the main reason that discouraged people from seeking help, and this inevitably led to an increasing suicide rate in the country [39].

Miller *et al*. [40] further explained the seasonal pattern could be shifted in the modern world due to the advancement in technology that enabled communication and improved connectivity, and thus reduced social isolation in between people. Nowadays, the social media together with internet using and many real-time communication tools like WhatsApp make connection with others much more available, accessible and affordable. Apparently, the new order of connectivity is much more frequent and common than ever [15]. Technology enhancement somehow reduces the temporal fluctuation of social interaction due to some traditional seasonal festivals and such changes in the practice of social activities may help explain the recent diminishing seasonal pattern of suicides in South Korea. Being one of the most connected online markets, the internet penetration in South Korea was extremely high with the rate of internet access per household reaching 99.5% in 2017 [41]. However, the Korean elderly aged 60+ seemed being isolated by the technology advancement and, by compared with other age groups, a relatively lower internet usage rate was observed [41]. Merely one fifth of older adults would access the internet in 2009 and the situation kept improving these days with the internet usage rate growing to 39.5% in 2015 and further to a rate of 58.8% in 2017 [41].

Higher incidence of suicide was reported among the old adults in summer time from the months of May to August while the winter season fewer suicides occurred [42–44]. Yip *et al*. [42] suggested that changing in living condition, the roles of males and females, and the mode of communication may somehow help reduce the seasonal impact towards suicidal behavior. In South Korea, the suicide rate among the elderly was high and the increase was noted far greater in rural regions [19]. In contrast to previous studies, two cycles per year was observed among the Korean elderly with a season peak in May followed by a second peak in October. However, the phenomenon of autumn peak was still far from conclusive [7].

Cowgill and Holmoes in 1970s [45] studied the status of older people in modern times and highlighted the modernization of a society lead to dramatic social changes that without doubt would affect the role of older people. The advanced health technology accounted for a higher life expectancy. According to the WHO published data in 2015, the total life expectancy in South Korea was 82.3 on average that ranked the eleventh in the World Life Expectancy [46]. Yet, a higher life expectancy implied an increase in the proportion of elderly in the population and introduced the concern of resources competition. In fact, the aging of South Korean population brought a most concern among OECD members [47] and the projected number of people aged 65 or older is expected to reach 15.6 percent of the total population in 2020 [48]. In addition, the breakdown of the traditional extended family that accompanied with urbanization of young generation also influenced the social status of older people [19]. Through a strong economic growth in South Korea over the past few decades, the financial hardship was prevalent among the elderly that nearly half of the population living below the poverty line [19, 49–50]. The issue of Korean elderly suicide is complex and deep-rooted that could not solely be explained. The observed seasonal pattern among the Korean elderly in our study becomes crucial evidence for instituting preventive measures, especially for identifying those high-risk groups and better resources planning on these days that are most needed.

Park *et al*. [47] compared the trends in suicide rates and methods among older adults across South Korea and Japan where both countries shared similar social contexts and demographic transitions. It was noted that a well-developed social welfare system for the elderly seems becoming one of key protective factors that prevented older adults from killing themselves. Improving social activities of this subgroup may help promote connectivity and reduce the impact of seasonality arising from improving of number of means of staying in contact. A better social welfare system together with pro-active engagement with older adults would certainly help to improving current situation.

## Limitation

Our study covered time series data of suicides from 1991 to 2015 in South Korea while the time the country experienced several economic downturns and a surge in the number of suicides was reported under such unfavorable economic conditions [51]. Economic factors may play a role in explaining the strong seasonal effect of suicide in the country. In addition, the effect of holiday was acknowledged to be one of common confounding variables in explaining the seasonality of suicide [13, 52–53]. A dip and peak pattern around major public holidays could be found among the Korean suicides [54]. It was noting that changing in season to the periods of year always associated with different social activities or traditional festivities. It was not easy to completely distinguish the potential impact of these two factors from the analysis of seasonal pattern in suicide mortality [13]. In our study, we confirmed the seasonal variation of suicides by adjusting the calendar effect and the findings can make directly comparison with those studies using the method of harmonic analysis [7, 10–11, 15]. It would be of future interest to explore the association in between the seasonality and those leading factors on the time series suicide data in South Korea.

Harmonic model has frequently been used to study cyclical pattern in epidemiological studies. A greater range of monthly variation of suicides was observed in the recent time intervals in South Korea and this made the modeling more challenging. The reduction in the proportion of seasonal component to the total variance may not be solely due to the diminishing in seasonal behavior of suicides, but the inadequacy of modeling to stochastic the cyclical variation. This clearly needs more research.
